# *k*-Space Hyperspectral Imaging by a Birefringent Common-Path Interferometer

**DOI:** 10.1021/acsphotonics.2c00959

**Published:** 2022-10-27

**Authors:** Armando Genco, Cristina Cruciano, Matteo Corti, Kirsty E. McGhee, Benedetto Ardini, Luca Sortino, Ludwig Hüttenhofer, Tersilla Virgili, David G. Lidzey, Stefan A. Maier, Andrea Bassi, Gianluca Valentini, Giulio Cerullo, Cristian Manzoni

**Affiliations:** † Dipartimento di Fisica, Politecnico di Milano, Piazza Leonardo Da Vinci 32, 20133 Milano, Italy; ‡ Istituto di Fotonica e Nanotecnologie-Consiglio Nazionale delle Ricerche, Piazza Leonardo Da Vinci 32, 20133 Milano, Italy; § Department of Physics and Astronomy, University of Sheffield, Hounsfield Road, S3 7RH Sheffield, U.K.; ∥ Chair in Hybrid Nanosystems, Nanoinstitute Munich, Faculty of Physics, Ludwig-Maximilians-Universität München, 80539 Munich, Germany; ⊥ School of Physics and Astronomy, Monash University, Clayton, Victoria 3800, Australia; # Department of Physics, Imperial College London, London, SW7 2AZ, U.K.

**Keywords:** *k*-space, hyperspectral imaging, optical microcavity, birefringent interferometer, metasurface

## Abstract

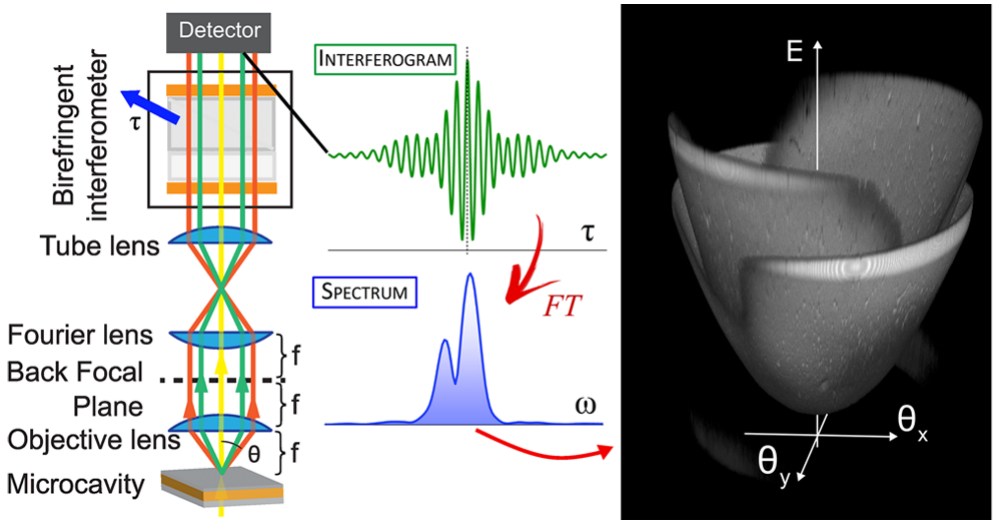

Fourier-plane microscopy is a powerful tool for measuring the angular optical response of a plethora of materials and photonic devices. Among them, optical microcavities feature distinctive energy-momentum dispersions, crucial for a broad range of fundamental studies and applications. However, measuring the whole momentum space (*k*-space) with sufficient spectral resolution using standard spectroscopic techniques is challenging, requiring long and alignment-sensitive scans. Here, we introduce a *k*-space hyperspectral microscope, which uses a common-path birefringent interferometer to image photoluminescent organic microcavities, obtaining an angle- and wavelength-resolved view of the samples in only one measurement. The exceptional combination of angular and spectral resolution of our technique allows us to reconstruct a three-dimensional (3D) map of the cavity dispersion in the energy-momentum space, revealing the polarization-dependent behavior of the resonant cavity modes. Furthermore, we apply our technique for the characterization of a dielectric nanodisk metasurface, evidencing the angular and spectral behavior of its anapole mode. This approach is able to provide a complete optical characterization for materials and devices with nontrivial angle-/wavelength-dependent properties, fundamental for future developments in the fields of topological photonics and optical metamaterials.

## 1 Introduction

Fourier-plane microscopy gives access to the in-plane momentum space (*k*-space) of the light coming from objects on the microscale.^1,2^ A way to access the *k*-space information is to measure the full electric field from the sample and subsequently to take the spatial Fourier transform. This method hence requires the knowledge of the electric field at the object plane, in both amplitude and phase. While amplitude can be obtained directly from the recorded intensity profile, the measurement of the spatial phase is much more challenging. To this aim, a variety of techniques have been introduced, such as TERMITES,^3^ SEA-TADPOLE,^4^ INSIGHT,^5^ and many more.^6^

An alternative method to access the *k*-space information is by exploiting the propagation of a field through a lens. In fact, when an object is placed in the focus of a lens, the spatial Fourier transform of its field is formed at the lens back-focal plane.^7^ The *k*-space intensity profile can therefore be obtained by imaging on a two-dimensional (2D) detector of the back-focal plane, instead of the object plane. In such a technique, the information about the angular distribution of the light field is extracted by imaging the objective back-focal plane on a two-dimensional detector, via a suitable optical system.^8^ This all-optical imaging approach gives high angular resolution over a wide field of view, but typically without providing any spectral information, since it integrates the light intensity over a broad wavelength range. On the other hand, multi- or hyperspectral imaging techniques^9,10^ are able to record, respectively, a discrete or continuous spectrum of the transmitted/reflected/emitted light for each point of the measured Field Of View (FOV) of the sample. All of the acquired spectra generate the so-called spectral hypercube, i.e., a three-dimensional (3D) data set of the intensity as a function of two spatial and one spectral (energy) coordinates. From the hypercube, it is possible to extract one image at a single spectral component or one spectrum at a single point of the image. Hyperspectral microscopes working in real space are extensively exploited in a wide range of fundamental and applied disciplines, e.g., biology/medicine,^11^ cultural heritage,^12^ and materials science.^13^

Spectrally resolved imaging is typically implemented either by placing spectral filters with fixed bandwidth before the detector^14,15^ (multispectral imaging) or by measuring the full spectrum of each pixel upon insertion of a dispersive spectrometer in the imaging system (hyperspectral imaging). The hypercube is then acquired by raster scanning the sample point by point^16^ (whisk-broom) or line by line^17^ (push-broom). The latter approach is commonly used in *k*-space confocal microscopy for the analysis of optical planar microcavities, which present nontrivial angle-dependent optical properties.^18^ In fact, the peculiar energy-momentum dispersion of photons confined in microcavities is key for a broad range of applications and phenomena, from the generation and manipulation of coherent light^19^ to the production of nonlinear optical devices^20^ and the observation of Bose–Einstein condensates in the solid state^21,22^ or optical analogues to spin–orbit interaction effects.^23^

More in detail, with the push-broom approach, one line of the *k*-space image is spatially selected by the entrance slit of a dispersive spectrometer placed before a two-dimensional detector, which acquires an energy-momentum cross-section of the 3D cavity dispersion. Then the *k*-space image of the sample formed on the slit is moved laterally to scan the following slices, thus sequentially recording the complete energy-momentum hypercube.^24^ This imaging technique provides an optimal energy resolution; however, the intrinsically high losses introduced by the spatial filtering and the dispersive elements impose long acquisition times. Therefore, to record the spectral information of the entire *k*-space of the sample with high angular resolution, tedious and alignment-sensitive scans are needed.

In this work, we combine the concepts of Fourier transform (FT) spectroscopy^25^ and momentum space (*k*-space) microscopy to demonstrate an innovative approach to wide-field and angle-resolved hyperspectral imaging and apply it to the characterization of the photoluminescence (PL) emitted by planar organic microcavities. Our method is based on a high-throughput common-path birefringent interferometer, which generates two delayed replicas of the (*k*-space) image, whose interference is measured as a function of their delay in a sequence of images. The FT of the resulting interferogram in each point of the imaged *k*-space yields the intensity spectrum for the corresponding coordinate within the angular field of view of the microscope. We apply our method to the characterization of the photoluminescence (PL) emitted by planar organic microcavities, revealing the parabolic dependence of the cavity modes energy with respect to the emission angle. From the hyperspectral image, we reconstruct a 3D image of the paraboloid, fully characterizing the cavity dispersion across the whole momentum space and as a function of polarization. We also apply the same method to a GaP nanodisk metasurface.

## 2 Results and Discussion

### 2.1 Hyperspectral *k*-Space Microscopy

**Figure 1:**
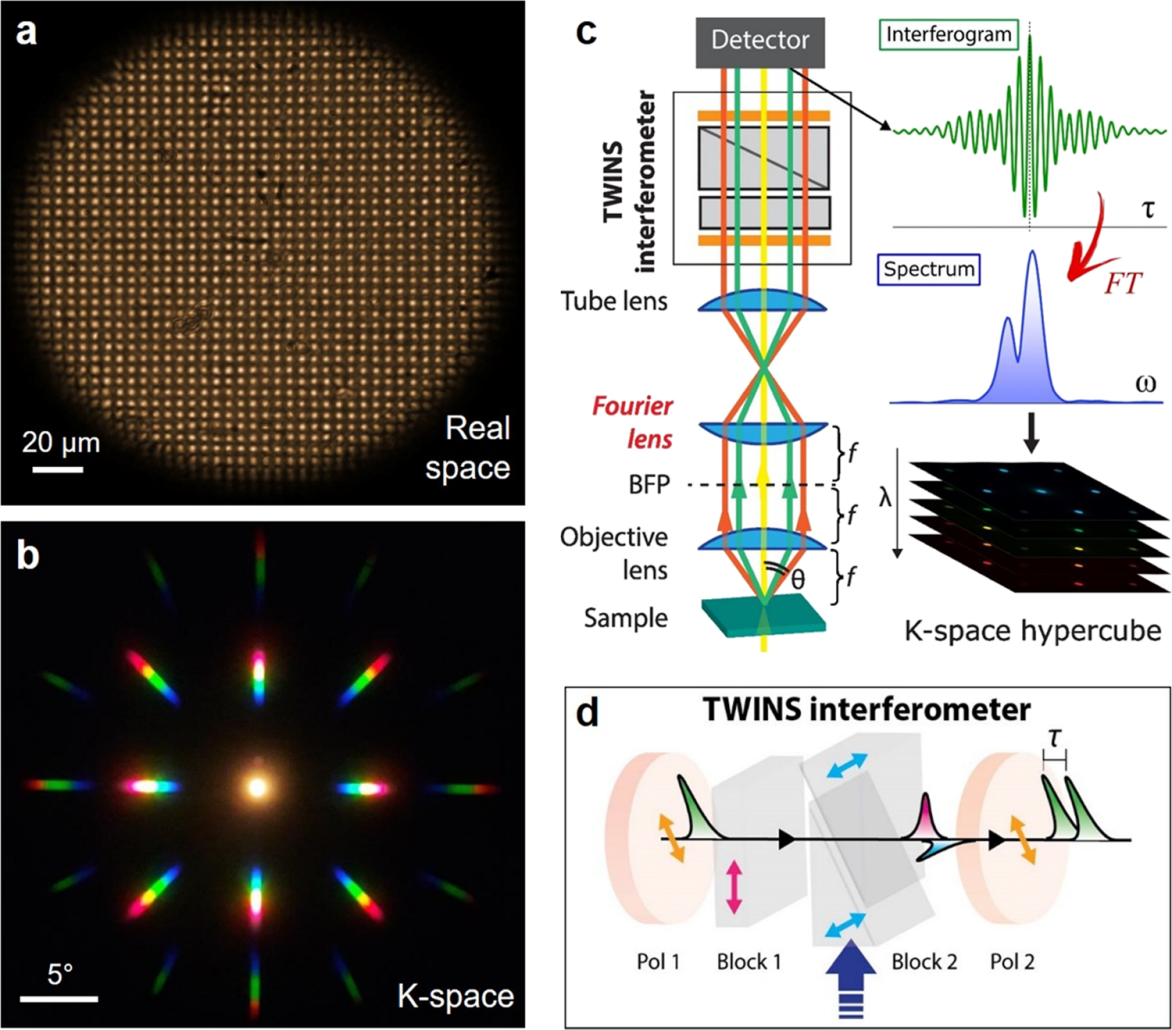
(a) Real-space microscope image of a 2D diffraction grating. (b) Image of the same grating in *k*-space showing the wavelength-dependent diffraction of a nearly collimated illuminating white light beam. (c) Layout of the *k*-space hyperspectral microscope, consisting of an objective lens, a Fourier (Bertrand) lens, a tube lens, the TWINS interferometer and a monochrome 2D detector. Varying the delay between the image replicas in the TWINS, each pixel records an interferogram, whose FT yields a spectrum for each element of the angular field of view. The Fourier space hypercube is then generated in a postprocessing step. BFP, back-focal plane. (d) Sketch of the common-path interferometer (TWINS) employed in our work.

Micro- and nanostructured optical materials often present a nontrivial angular response to transmitted, reflected, or emitted light. A prototypical example of such devices is a diffraction grating, which reflects or transmits light at different angles depending on its wavelength. The diffraction angle depends on the periodicity of the patterns at the grating surface, following Bragg’s diffraction law. A standard real-space microscope can characterize the micromorphology of such device, measuring the spatial period of the grating, or its uniformity across the FOV, but fundamental information about the angular properties of the diffracted light are inherently lost. In fact, the intensity recorded for each point of the image is a sum of the contributions of the light rays from different angles collected by the objective lens within its Numerical Aperture (NA). Figure 1a shows a real-space microscope transmission image of a transparent 2D plastic grating made of two orthogonal sets of slits with a period of 200 lines/mm The measurement is performed illuminating the sample with broadband incoherent light from a lamp and collecting the transmitted light with the microscope objective. The tips of the periodic pyramidal pattern appear illuminated, while darker regions show some defects and inhomogeneities, but no wavelength-dependent patterns can be noticed, and no information can be inferred concerning the propagation direction of the transmitted light.

A way to discern the angular dependence of the light spectrum is to project an image of the objective back-focal plane on a camera,^2^ obtaining a *k*-space view of the grating (Figure 1b), where the *x*–*y* coordinates now represent the angular coordinates. The objective back-focal plane is imaged into the detector by placing a Bertrand (or Fourier) lens before the tube lens, as shown in Figure 1c. An infinity-corrected objective, like the one used in our microscope, needs additional lenses to project the image of the analyzed sample on the detector. Working in a standard configuration for real-space imaging, typically only one lens is placed after the objective, called tube lens, which forms a magnified image of the sample at a distance equal to its focal length, where the camera is placed. The Fourier-transformed image of an object is instead automatically formed behind the focusing lens on a virtual plane called the back-focal plane or Fourier plane. Therefore, to image the *k*-space of our sample on the detector, one more lens, called Fourier lens, must be placed at a distance equal to its focal length from the back-focal plane of the objective. The white spot at the center of the image represents the zero-order diffraction of the collimated illumination beam. Since it is perpendicular to the sample plane, the white spot is at coordinates θ_*x*_ = 0 and θ_*y*_ = 0. Two orders of diffractions are clearly visible in distinct regions around the central spot. The rainbow within each lateral stripe appears as a direct consequence of Bragg’s law, presenting diffracted colors going from blue to red with increasing angle. More complex samples feature even richer wavelength- and angle-dependent optical properties, motivating the need of a novel hyperspectral technique tailored for *k*-space microscopy.

To add spectral resolution to the microscope and to produce hyperspectral images of the *k*-space, we exploit FT spectroscopy. In this technique, the optical signal from the sample is split into two replicas that are mutually delayed, recombined, and finally sent to a detector. The interference between the two replicas, recorded as a function of their relative delay, gives rise to an interferogram. According to the Wiener–Khinchin theorem, the FT of the interferogram yields the intensity spectrum of the optical waveform.^26^ Using a 2D detector matrix, the technique can be easily parallelized and extended to an imaging system, enabling the measurement of the spectrum for each pixel of the image within the angular FOV (Figure 1c).

FT hyperspectral imaging has important advantages with respect to standard dispersive multi/hyperspectral methods, such as a higher signal-to-noise ratio in readout-noise-dominated systems, higher throughput (no spatial filters are present in the detection path^27,28^), and flexible spectral resolution adjusted by setting the maximum delay of the acquired interferogram. Most FT hyperspectral imaging configurations generate the replicas using Michelson or Mach–Zehnder interferometers,^29^ which are highly sensitive to vibrations and require active stabilization approaches to reach a sufficient stability. In our setup, instead, we use a common-path birefringent interferometer, the Translating-Wedge-based Identical pulses eNcoding System (TWINS).^30^ A scheme of our TWINS interferometer is displayed in Figure 1d. Block 1 (B1) and Block 2 (B2) are two birefringent crystals made of α-barium borate (α-BBO), while Pol1 and Pol2 are two broadband polarizers (working range 400–2000 nm) with high extinction ratio (>5000).

Pol1 polarizes the input light at 45° with respect to the optical axes of B1 and B2. B1 is a plate that introduces a fixed phase delay between the two orthogonal polarizations that propagate along the fast and slow axes of the material. B1 and B2 have orthogonal optical axes; thus, they introduce a delay of opposite sign between the two polarizations. B2 is shaped in the form of two wedges and one wedge is put on a motorized translation stage to finely control the delay between the two replicas within a small fraction (approximately 1/100) of the optical cycle (e.g., 20 attoseconds for visible light with a wavelength λ = 600 nm, which has an oscillation period of 2 fs). The second polarizer is set at 45° with respect to the optical axes of B1 and B2 and projects the two fields back to the same polarization, ensuring interference between the two replicas. Recently, the TWINS has been employed as a FT spectrometer with both coherent and incoherent light beams,^31,32^ in different photon energy ranges from the visible to the mid-infrared. TWINS has static delay fluctuation lower than 1/360th of the optical cycle,^26^ enabling long-exposure measurements. The spectral resolution of a FT spectrometer depends on the range of the delay scan introduced by the interferometer, which in TWINS depends on the birefringence of the plates, their travel range, and the tip angle. The best spectral resolution of the presented device, corresponding to the longest scan, is 3 THz (4 nm at λ = 635 nm), while the minimum measurable spectral shift was found to be less than 0.1 nm; finally, the measured background noise is −30 dB.^25^

The interferometer is compact (<10 × 10 cm^2^ footprint), with exceptional static and dynamic delay stability in the visible spectral range.^30^ The small footprint allows placing the instrument between the tube lens of a microscope and an external camera (see Figure 1c), without modifying the internal optical path and avoiding any vignetting. In addition, the interferometer preserves the angular resolution of the microscope, being about 0.3° in our case. This broadens the applicability of our approach to *k*-space hyperspectral imaging to almost any commercial microscope equipped with a Bertrand lens. More details about the experimental setup, i.e., the optimized positioning of the interferometer, the camera performances, frequency calibration, and resolution, can be found in refs (25, 33). Our system enables high-quality wide-field hyperspectral imaging, generating spectral hypercubes containing a full spectrum for each pixel.

### 2.2 *k*-Space Hyperspectral Imaging of Planar Microcavities

**Figure 2:**
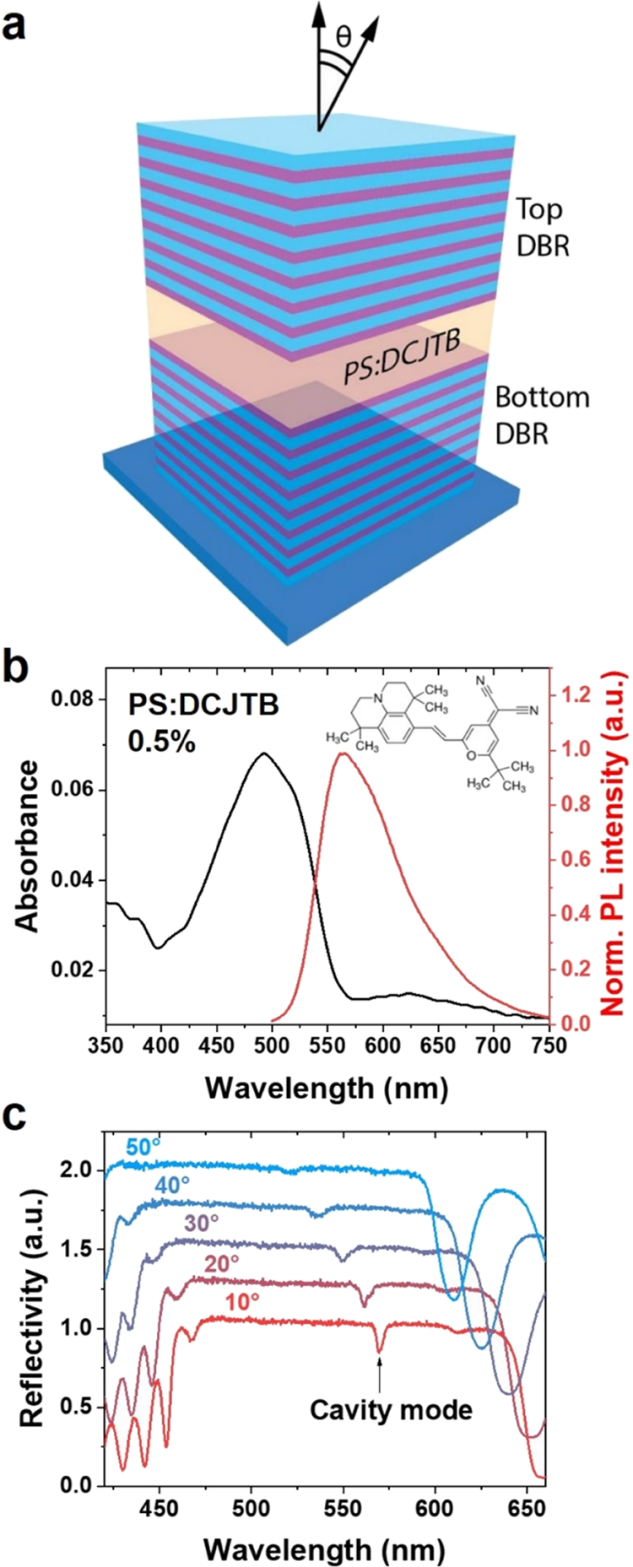
(a) Schematic of a planar microcavity consisting of a polymeric spacer (PS) doped with fluorescent molecules (DCJTB) sandwiched between two distributed Bragg reflectors (DBRs). The viewing angle θ is defined as the angle between the cavity axis, normal to the sample plane, and the viewing direction. (b) Absorption (black line) and PL (red line) spectra of DCJTB. Inset: DCJTB molecular structure. (c) Stacked plot of the reflectivity spectra measured at different angles for a microcavity with a DCJTB concentration of 0.5% in the PS matrix, showing the cavity mode formed within the DBRs’ stopband, blue-shifting with increasing θ.

Our technique is ideally suited to test planar optical microcavities, which feature prominent angle-dependent properties. Such optical resonators are arranged in a vertical architecture consisting of two highly reflective mirrors distanced by a spacer. Figure 2a shows the structure of the devices employed in this work, which consist of two distributed Bragg reflectors (DBRs), each made of several pairs of dielectric layers (SiO_2_/TiO_2_), distanced by a polymeric spacer. The bottom and top DBRs are composed of 10.5 and 8 pairs, respectively. The optical length for each layer is n*L = λ_0_/4, where *L* is the layer thickness, *n* is the refractive index, and λ_0_ is the central wavelength of the high reflectivity spectral window of the mirror (stopband). A blend of a transparent polystyrene (PS) polymeric matrix doped with a bright fluorescent molecule (DCJTB)^34^ forms the cavity spacer.

The PL and absorption spectra of the organic film are shown in Figure 2b together with the molecular structure of the fluorescent dye. DCJTB shows a broad PL, peaked at about 600 nm with a tail at longer wavelengths, and an absorption peak at about 500 nm. Such a large Stokes shift prevents the reabsorption of emitted photons, being beneficial for the cavity emission efficiency. The presence of a spacer between the mirrors creates a defect in the one-dimensional (1D) photonic periodic structure, leading to a resonant optical mode created by the constructive interference of electromagnetic waves traveling inside the cavity. This cavity mode results in a dip in the reflectance spectra (Figure 2c), whose spectral position depends on the distance *L*_c_ between the mirrors. Due to the strong light confinement along the direction orthogonal to the cavity plane (cavity axis), the angular dispersion of the cavity mode energy *E*_cav_(θ) (wavelength λ_cav_(θ)) increases (decreases) with the viewing angle θ, linked to the in-plane photon momentum *k*_∥_ = *k *sin(θ), following a semiparabolic trend.^21^

**Figure 3:**
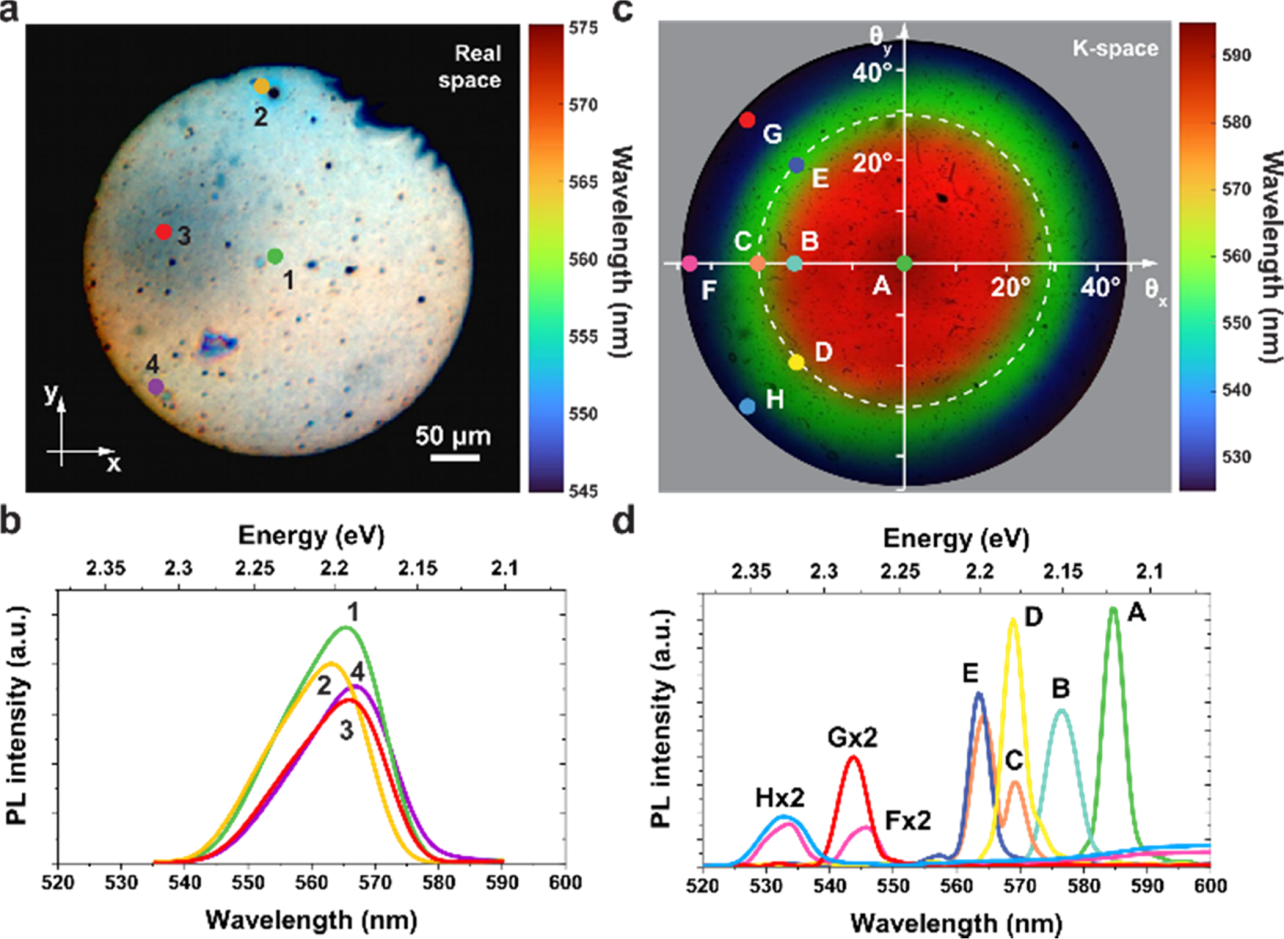
(a) False-RGB image extracted from the hyperspectral PL measurement of the PS:DCJTB microcavity in real space. The colored circles show the points whose spectrum is plotted in panel (b). (b) PL spectra of four points extracted from the real-space hyperspectral measurement of the microcavity. (c) False-RGB image generated from the hyperspectral PL measurement of the PS:DCJTB microcavity in *k*-space, plotted in angular coordinates. The qualitative false-RGB color images are obtained by band-pass filtering the spectra in three adjacent spectral regions, assigned to the R, G, and B channels, respectively. The scale bar is related to the resulting color assignment. The colored circles refer to the points whose spectrum is displayed in panel (d). The white dashed line represents an iso-angle ring at about 30°. (d) PL spectra of the colored spots extracted from the *k*-space hyperspectral measurement of the microcavity (c). Spectra F, G, and H are multiplied by a factor of 2.

As a reference for our hyperspectral experiments, we performed angle-dependent reflectivity measurements on a cavity with 0.5% of DCJTB in PS (Figure 2c) using a standard optical setup equipped with a goniometer to rotate the viewing angle with respect to the cavity axis (see details in the Supporting Information). At 10°, the cavity mode reflectivity dip is at 570 nm, within the DBR stopband spanning between ≈460 and 650 nm. The mode gradually broadens and blue-shifts reaching 520 nm at 50°. It is worth noting that this spectroscopic method for characterizing the microcavity presents strong disadvantages: the angular resolution is typically quite low and acquiring spectra at small angles is very challenging. The resolution can be improved in principle using a long goniometer arm, but at the price of decreasing simultaneously the signal intensity, thus requiring long scans with small angular steps. We tested the performance of our hyperspectral microscope by measuring the PL emitted by the same cavity in both real space and *k*-space. The former configuration consists of a simple microscope architecture with a 20X objective lens and a tube lens in the detection path, with the TWINS interferometer placed between the tube lens and the CCD camera. The cavity was illuminated by a UV laser (365 nm) coupled to a multimode fiber, whose output tip flat-field was imaged on the sample using a collimation lens and the same objective used for the detection. A dichroic mirror was used to collect the PL emitted by the microcavity, with a long-pass filter rejecting the illumination light (more technical details about the hyperspectral microscope in the real-space configuration are reported in the Supporting Information). The interferometer delay scan allows the camera to record the interferograms and, after FT, to obtain the corresponding intensity spectra for each pixel (∼1Mpixel in total). The resulting spectral hypercube is transformed in the false-RGB image shown in Figure 3a, obtained by mapping the visible spectrum in the range between 545 and 575 nm. The hyperspectral image in real space already provides rich information about the sample morphology. Specifically, it appears that the device area is not as homogeneous as expected. Regions of different colors across the sample indicate variations in the PL spectrum. One reason is that the thickness of the organic spacer varies in the area surrounding the internal impurities, leading to a modification of the cavity resonant wavelength. Dark spots on the analyzed region are related to defects and impurities present either on the surface of the top mirror or between the mirrors. In the latter case, the inclusion of small dirt particles might have occurred during the growth process of the organic spacer, deposited via spin-coating.

We extracted the PL spectrum of four points selected across the hyperspectral image, plotted in Figure 3b. The peak wavelength of the PL spectra extracted from different points changes slightly compared to the central spot (565 nm, green circle in Figure 3a), spanning from 563 to 566 nm. The yellow spectrum in Figure 3b is blue-shifted compared to the one in the central spot, being related to the area surrounding an internal defect with a lower cavity thickness (yellow circle in Figure 3a). Resolving such small spectral variations with this level of spatial detail can be very challenging using other conventional imaging methods. All of the spectra are broader compared to the cavity modes measured in reflectivity, showing in particular a prominent short-wavelength tail. In real-space measurements, in fact, the PL spectrum of each pixel is a convolution of the cavity mode spectra at different angles collected within the objective NA. Hence, the observed broadening toward shorter wavelengths can be explained considering the blue-shifting cavity dispersion with increasing angle.

To perform *k*-space hyperspectral imaging of the microcavity, we placed the Bertrand lens in the optical path of the microscope, focusing on the back-focal plane of a 100X objective (NA 0.75), between the objective lens and the dichroic mirror. For the excitation, we used the same fiber-coupled laser employed for the real-space experiments. A false-RGB image created from the PL hyperspectral datacube in *k*-space is shown in Figure 3c as a function of the angular coordinate. The angular FOV spans between 48 and −48°, being only limited by the NA of the objective lens. The calibration procedure for converting the camera pixels to angles to produce the axes of the *k*-space image is reported in the Supporting Information. At variance with the real-space image, here, a strong variation of the PL signal occurs across the viewed angles. Starting from the center of the image at zero angle, the peak wavelength gradually decreases going toward the periphery of the angular FOV. The hyperspectral micrograph shows an elliptical shape with the major (minor) diameter aligned with the diagonal (antidiagonal) of the image. We will clarify the reason of this asymmetry below.

The spectra extracted from the hypercube present very interesting features (Figure 3d). The cavity mode at the normal direction θ_*x*_ = θ_*y*_ = 0 (point A, green spot in Figure 3c) shows a very narrow spectrum (green curve in Figure 3d), peaked at 583 nm, with a linewidth similar to the one measured in reflection using a goniometer setup (Figure 2c). For angles from 0 to 20°, the peak wavelength blue-shifts and broadens quite regularly (point B, turquoise spot in Figure 3c and spectrum in Figure 3d). For larger angles along the horizontal axis (point C, orange spot), the spectrum blue-shifts more rapidly and splits into two clearly resolved peaks. Moving further from this point along an iso-angle circle (white dashed line in Figure 3c) toward the diagonal and antidiagonal axes, either the short wavelength or the long wavelength peak are, respectively, suppressed, resulting again in single cavity modes (yellow (D) and dark blue (E) spectra in Figure 3d). This effect is even stronger at the extreme angles of our measurements (about 48°), where the pink spot (F) on the horizontal axis of Figure 3c shows two peaks far apart from each other (pink spectrum (F) in Figure 3d), both strongly blue-shifted compared to the spectrum at zero angle. Also, in this case, moving toward the diagonal and antidiagonal axes (red (G) and blue (H) spots), the modes at short and long wavelengths are, respectively, suppressed.

### 2.3 Analysis of the Cavity Dispersion

The origin of the two distinct peaks at symmetric directions D–E and G–H can be ascribed to the presence of transverse electric (TE) and transverse magnetic (TM) modes with different dispersions. This property is specific to planar microcavities made of dielectric mirrors and stems from the different penetration depths into the DBRs for light with orthogonal polarization.^35,36^ The high angular and spectral resolution of our *k*-space microscope allows us to easily produce a 3D reconstruction of the hypercube as a function of the photon energy and the angular coordinates, which helps to understand the cavity behavior for different polarizations.

**Figure 4:**
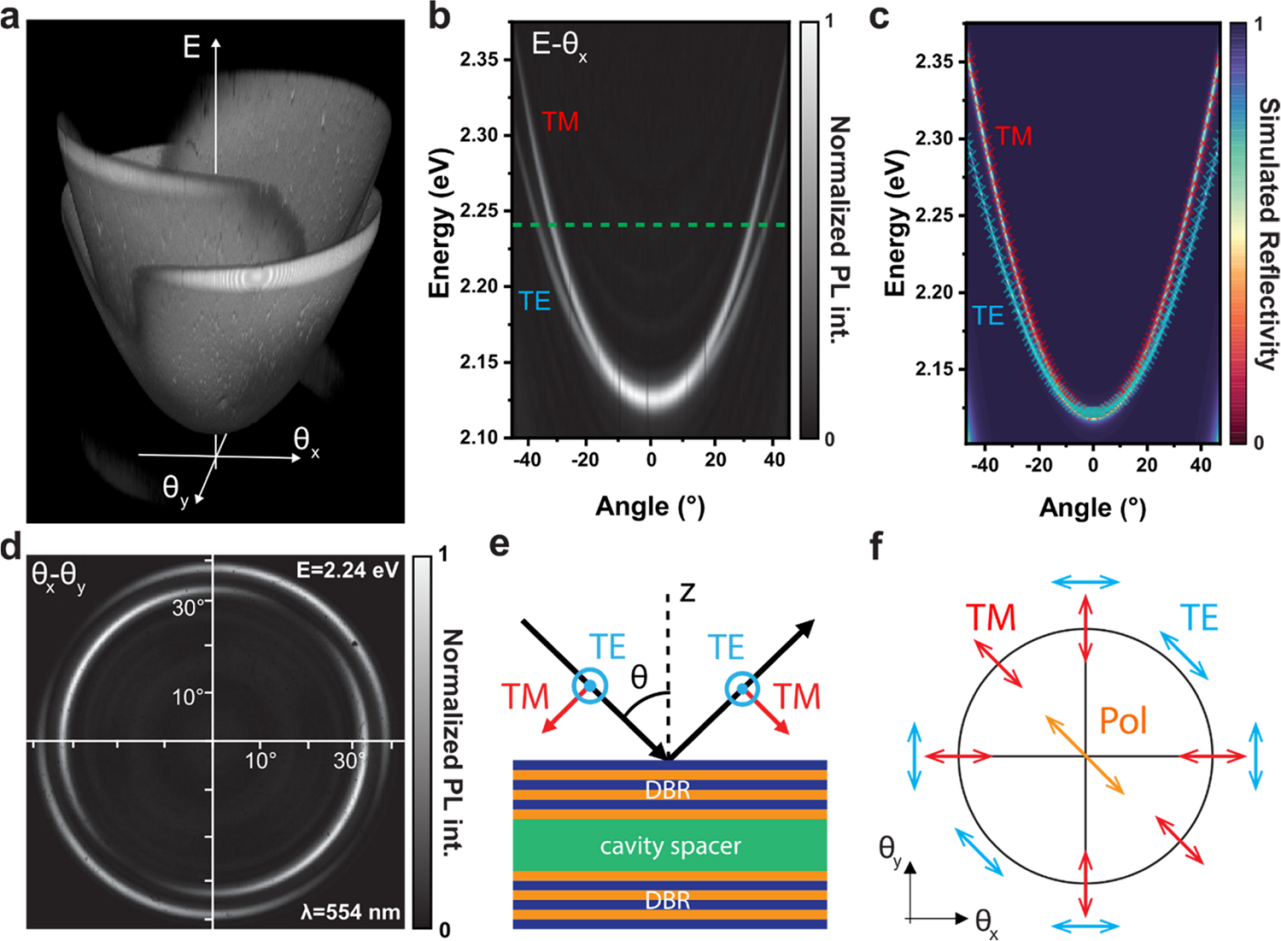
(a) 3D cavity dispersion reconstructed from the *k*-space PL hyperspectral image of the PS:DCJTB microcavity, displayed in the energy-angles space. The 3D image is shown as texture-based volume rendering. (b) Vertical cross-section of the 3D hyperspectral image showing the parabolic dispersions and the TE–TM splitting for the microcavity. (c) Color map of the simulated reflectivity for the analyzed microcavity, with the overlapped experimental cavity modes energies (red and blue crosses). (d) Horizontal cross-section of the 3D hyperspectral cavity data taken at *E* = 2.24 eV (λ = 554 nm, green dashed line in (b)). (e) Sketch of the configuration for TE and TM polarized light incident to the cavity. The black arrow represents the propagation direction of light, while the blue and red vectors display the electric field in the different polarizations. (f) Schematics of the polarizations selected by the TWINS polarizer (Pol1) across the iso-angle ring of the cavity dispersion in the θ*_x_* – θ*_y_* plane.

The 3D image in Figure 4a maps the peak energy of the cavity PL in the *k*-space datacube as a function of angles θ*_x_* and θ*_y_*. The image shows two compenetrated TE and TM paraboloids, which gradually vanish at high energies and only for certain angles, related to the points of Figure 3c, where only one mode is visible. The external paraboloid appears rotated by 90° compared to the internal one. Figure 4b shows a vertical cross-section of the 3D image, taken along the θ*_x_* axis, where the TE–TM splitting is clearly visible. The resonance mode frequency (ω_m_ = *E*_cav_/*h*) is dependent on the effective cavity length (*L*_eff_ = *L*_c_ + *L*_DBR_), taken as the sum of the cavity spacer thickness (*L*_c_) plus the EM field penetration depth into the DBRs (*L*_DBR_), the latter being dependent on both angle and polarization of the incident light
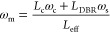
1where ω_s_ is the central frequency of the stopband and ω_c_ is the Fabry–Perot cavity mode frequency.^37^ When ω_c_ ≠ ω_s_, the TE–TM splitting becomes angularly dependent, increasing with the ω_s_ – ω_c_ difference
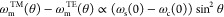
2In our case, the TM (TE) mode can be identified as the high (low) energy branch, considering that ω_s_ is higher than ω_c_. We modeled the TE and TM modes dispersion using the transfer matrix method, which can accurately simulate the complex optical structure of our cavity (see details in the Supporting Information). Figure 4c shows that an almost perfect match is obtained by comparing our data with the theoretical reflectivity.

Horizontal cross-sections of the 3D hyperspectral image show perfectly concentric rings, splitting at high energies into the TE and TM modes. Figure 4d shows an example of horizontal cross-section, taken at 2.24 eV (λ = 554 nm, green dashed line in Figure 4b), where the TM (TE) mode intensity gradually decreases along the ring going toward the diagonal (antidiagonal) direction, before increasing again. This behavior can be explained considering that the TE polarization of light incident on the cavity at an angle θ is always parallel to the surface of the cavity, while the TM polarization lies in the same plane of the *z*-axis, normal to the cavity surface (Figure 4e). As such, owing to the cylindrical symmetry of the device, the TE polarization is always tangential to the iso-angle rings in the horizontal cross-sections of the cavity paraboloid, while the TM polarization is always radial (Figure 4f). The Pol1 polarizer of the TWINS interferometer (orange arrow in Figure 4f) then selects only the polarization oriented at 45° for each point of the *k*-space, allowing the detection of only the TE (TM) mode along the diagonal (antidiagonal) direction or both modes equally along the vertical and horizontal directions. With our technique, three of the four Stokes parameters of a centrosymmetric microcavity could be in principle retrieved at every measured angle,^38^ specifically *S*_0_ = *A* + *D*, *S*_1_ = *A* – *D* and *S*_2_ = *H* – *V*, where *A*, *D*, *H*, and *V* are the PL intensity images taking, respectively, the antidiagonal, diagonal, horizontal, and vertical cross-sections of the cavity dispersion. To measure the fourth Stokes parameter, *S*_3_, further optimizations of the setup are needed, considering the implementation of a λ/4 retarder plate. As such, our method would allow the complete characterization of the polarization behavior of optical devices with isotropic in-plane properties by simple measurements.

### 2.4 Hyperspectral *k*-Space of GaP Nanodisks

**Figure 5:**
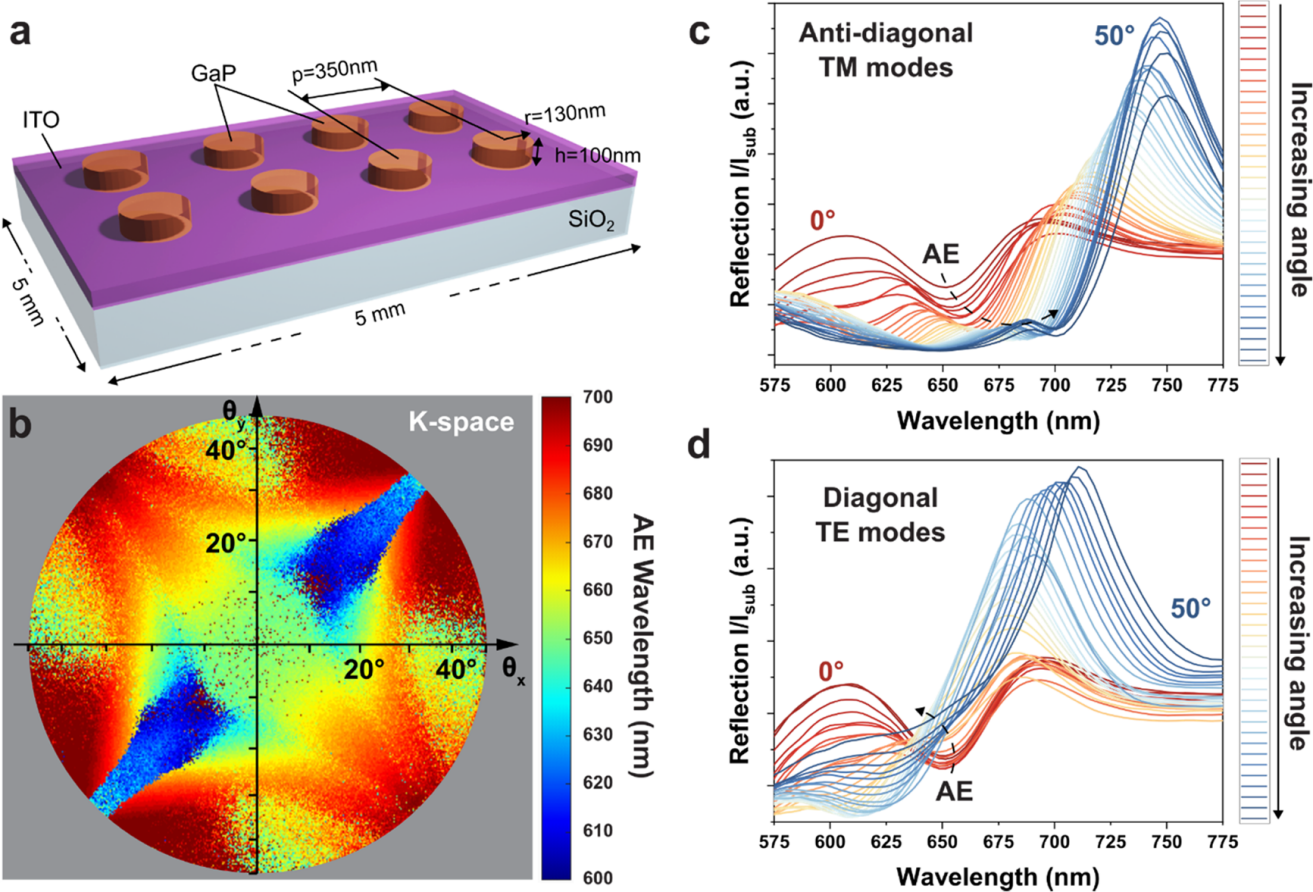
(a) Sketch of the GaP nanodisk metasurface fabricated on an ITO/SiO_2_ substrate by nanoimprinting. (b) Color map of the AE dip wavelength variation across the *k*-space, generated from the hyperspectral reflectivity measurement of the GaP nanodisks normalized by the substrate reflectivity, plotted in angular coordinates. (c, d) Reflectivity spectra (*I*/*I*_sub_) extracted from the hyperspectral *k*-space image, moving from the center (0°) along the (c) antidiagonal and (d) diagonal directions, associated, respectively, with the TM and TE polarized optical modes of the sample.

We further demonstrate the capabilities of our hyperspectral *k*-space imaging setup by measuring a metasurface of dielectric nanoresonators made of GaP nanodisks (diameter *d* = 260 nm) on an ITO/SiO_2_ substrate (Figure 5a). High-refractive-index semiconductor nanoresonators can support optical Mie resonances in the form of interference of electric and magnetic multipoles, such as the nonradiative anapole excitation (AE), which can lead to high light confinement within the resonator.^39−41^ Periodic arrays of resonators can also lead to lattice resonances (LRs), caused by the resonant coupling of dipolar modes of closely packed nanostructures.^42−44^ The employed sample exhibited a nonradiative AE as a dip in reflectivity at about 650 nm and a less prominent LR at shorter wavelengths. More details on the fabrication and the basic optical properties of this device can be found in our previous study.^45^ The resonant modes of such device are supposed to show nontrivial angular dispersions, which has not been studied yet.

For the *k*-space hyperspectral measurements, we illuminate the sample at all the angles within the objective NA using a broadband white light and record the reflected light intensity (a detailed sketch of the setup configuration is shown in Figure S3 in the Supporting Information). We point out that the nanodisk array is highly packed (array period *p* = 350 nm), so we can neglect any Bragg diffractive effect from the optical response of the sample, in the wavelength range of our interest. On the other hand, the nanoimprint lithography method allowed us to fabricate large area devices (5 × 5 mm^2^), much bigger than our illumination spot in real space, hence ensuring a uniform response. Figure 5b shows the hyperspectral *k*-space image of the nanodisks: the angular color map was reconstructed by plotting the AE dip wavelength extracted from the reflection spectra, normalized by the measurement on a flat GaP region, in the wavelength range between 600 and 700 nm (the raw maps are reported in Figure S7 in the Supporting Information). In this case, we use a 100X objective with NA = 0.8, inserting a linear polarizer in the excitation path, parallel to the analyzer in the TWINS. The image shows a clear variation of the reflectance spectra going from zero to higher angles, with different behaviors moving along the diagonal or the antidiagonal directions. The nanodisks are centrosymmetric resonators; hence, for the anapole mode, we expect a polarization pattern as previously shown in Figure 4f: the TE and TM modes are detected, respectively, along the diagonal and the antidiagonal directions of the *k*-space image.

We plot several spectra taken on the antidiagonal (diagonal) direction of the hyperspectral image in Figure 5c (d), as a function of the angle. The dark-red curve is related to the spectrum at 0°, which shows a clear dip at about 650 nm ascribed to the AE state.^45^ The origin of this feature was also demonstrated by our numerical finite-difference time-domain (FDTD) simulations (see Figure S4 in the Supporting Information), showing the electric field being confined in the nanodisk at the resonant wavelength. For TM polarization (Figure 5c), we observe a gradual red shift of the AE resonance upon increasing the angle up to 50°, which moves from ∼650 to ∼700 nm, in close agreement with the simulated behavior (see Figure S5 in the Supporting Information). The response radically changes for the TE mode (Figure 5d), which slightly blue-shifts, then gradually disappearing at high angles, matching again the simulated trend (see Figure S5 in the Supporting Information). It is worth noting that such a different angular behavior between TE and TM modes has been previously observed also in other dielectric nanodisks metasurfaces, being strongly affected by the perturbation of additional electromagnetic multipoles, besides the anapole modes.^46,47^

## 3 Conclusions

In summary, we developed a novel approach for *k*-space hyperspectral microscopy based on a common-path birefringent interferometer, capable of measurements on a wide angular FOV; the proposed method has high throughput, angular resolution of 0.3°, and spectral resolution of 3 THz (4 nm at λ = 635 nm), while the maximum resolution in terms of spectral shift is less than 0.1 nm. We applied our technique to the characterization of organic planar microcavities made of a fluorescent polymeric blend sandwiched between two dielectric mirrors, measuring the cavity dispersion in PL across the whole energy-momentum space. The TE–TM splitting of the cavity modes can be clearly observed, and the measured dispersion matches very well with theoretical simulations. Furthermore, we fully characterize the optical behavior of a dielectric nanodisks metasurface by measuring the hyperspectral reflectivity in *k*-space. The compact footprint and the superior stability of the interferometer allow an easy implementation in custom microscopy setups or even in commercial microscopes equipped with a Bertrand lens. With further optimization, it could be also used for measuring the frequency-dependent linear and circular Stokes parameters of the analyzed samples. The proposed method can serve as a fast and reliable characterization tool for a broad range of materials and devices with nontrivial angle/wavelength-dependent optical properties, such as 2D semiconductors^48,49^ and metasurfaces.^50^

## 4 Methods

For the hyperspectral setup, we used a commercial optical microscope (Leica DMRBE) equipped with an optional Bertrand lens, and a tube lens with a focal length of 250 mm. The light delivery path was modified with a fiber-coupling system designed to obtain a uniform light spot on the sample in epi-illumination configuration. The eyepiece block of the microscope was replaced by the TWINS interferometer mounted with a threaded support, ensuring its stability. Above the TWINS, in the same fashion, an EMCCD camera (Andor Luca*EM* R 1004 × 1002 pixels, 8 μm each, 14 bit) was mounted. For the excitation, we used a UV laser (365 nm) coupled to a multimode fiber. A filter cube reflects the input light to the sample and transmits the light emitted by the sample to the camera. More details can be found in the Supporting Information. The 2D grating is produced by Plymouth Grating Laboratory with scanning beam interference lithography. For the experiments shown in this work, the volume of the raw temporal datacube is typically about 1Gbyte. The Fourier transform of the whole datacube requires few tens of seconds with a standard commercially available PC (RAM: 16GB, CPU: Intel i7 or similar), while the resulting spectral hypercube also has a volume of about 1Gbytes.

## Supporting Information Available

The document contains details about the following: the goniometric setup for angle-resolved reflectivity measurements; the hyperspectral microscopy setup; the calibration of the angular FOV; the transfer matrix simulations; the microscope configurations used for the different experiments; the GaP nanodisks simulations; and the GaP nanodisks raw *k*-space maps (PDF)
