# Supplementary material for: k-Space Hyperspectral Imaging by a Birefringent Common-Path Interferometer
Source: ACS Photonics. 2022 Oct 27;9(11):3563–72. doi: 10.1021/acsphotonics.2c00959 (PMC9673149; doi:10.1021/acsphotonics.2c00959)
Supplement: Supplementary file 1 — ph2c00959_si_001.pdf [file ph2c00959_si_001.pdf]

Supplementary materials

K-Space Hyperspectral Imaging by a Birefringent Common-Path Interferometer

Armando Genco,[1*] Cristina Cruciano,[1] Matteo Corti,[1] Kirsty E. McGhee[3], Benedetto Ardini,[1] Luca Sortino,[4] Ludwig Hüttenhofer,[4] Tersilla Virgili[2], David G. Lidzey[3], Stefan A. Maier,[4,5,6] Andrea Bassi[1], Gianluca Valentini[1], Giulio Cerullo[1,2] and Cristian Manzoni[2**]

[1]Dipartimento di Fisica, Politecnico di Milano, Piazza Leonardo Da Vinci 32, 20133, Milano, Italy

[2]Istituto di Fotonica e Nanotecnologie-Consiglio Nazionale delle Ricerche, Piazza Leonardo Da Vinci 32, 20133, Milano, Italy

[3]Department of Physics and Astronomy, University of Sheffield, Hounsfield Road, S3 7RH, Sheffield, UK

[4] Chair in Hybrid Nanosystems, Nanoinstitute Munich, Faculty of Physics, Ludwig-Maximilians-Universität München, 80539 Munich, Germany

[5] School of Physics and Astronomy, Monash University, Clayton, Victoria 3800, Australia

[6] Department of Physics, Imperial College London, London, SW7 2AZ, United Kingdom

1. Goniometric setup for angle-resolved reflectivity measurements

Angle-dependent white light reflectivity measurements were performed using a goniometer setup consisting of two arms attached to a motorised rotation stage. A fibre-coupled Halogen-Deuterium white light source (DH-2000- BAL) was attached to the first arm and light was focused on the sample using a series of lenses. Reflected light was collected through a series of lenses mounted on the second arm and directed into a spectrometer (Andor Shamrock SR-303i-A CCD) using an optical fibre.

2. Hyperspectral microscopy setup

We used two different objectives for hyperspectral imaging:

- 20x HC PL FLUOTAR, Leica with N.A.=0.5 for real space measurements;
- 100x HC FLUOTAR, Leica with N.A.=0.75 for the k-space measurements.

The motor controller of the TWINS and the imaging camera are connected to a computer so that the user can control the interferometric scan steps and the camera parameters necessary for the measurement, through a LabVIEW software. For measurements in real space, the interferometer performed a delay scan from -90 fs to +90 fs, for a light component at 600 nm wavelength. In k-space, the delay scan spanned the whole excursion of the wedges, in order to reach the maximum spectral resolution (4 nm at lambda=635nm). The exposure time for PL measurements was 6s per step, while for reflection measurements it was 25 ms and 10 ms, for GaP nanodisks and substrate, respectively.

3. Calibration of the angular FOV

To calibrate the FOV we used the 2D grating shown in Figure 1 of the main text. Our goal was to find the angles for each order of diffraction, obtaining a correspondence with the camera's pixel when performing k-space imaging of the same grating. To this aim, we illuminated the grating at 0° with a collimated helium-neon (He-Ne, λ = $633nm$) and a green diode laser (λ = $532nm$), and projected the far-field diffraction pattern on a screen at a known distance. We then measured the position of the diffraction orders (up to the 3rd order) with respect to the 0th order, hence obtaining the following table for the diffraction angles:

	1st order	2nd order	3rd order
HeNe laser (633 nm)	7.4°	15°	22.9°
diode laser (532 nm)	6.12°	12.4°	18.9°

Finally, we obtained the calibration of the k-space image of the grating shown in Figure S1. From the calibration of the k-space imaging, we determined an angular pixel resolution of about 0.3°, taking also into account the camera pixels size and the total magnification of the angular FOV.

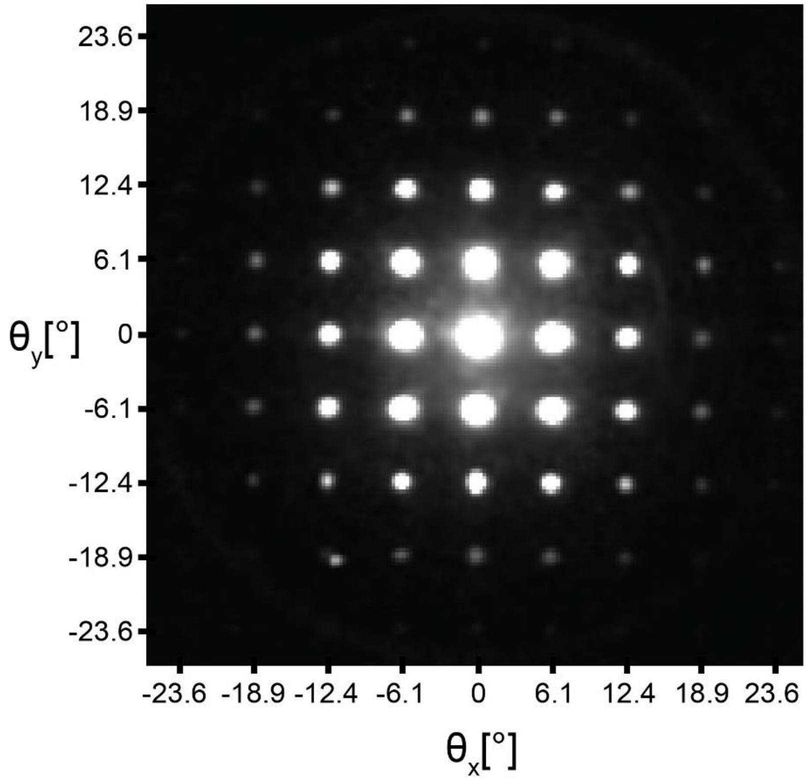

Figure S1. K-space image of the diffraction grating used to perform the angular FOV calibration with the 532 nm diode laser. The horizontal and vertical axes report the calibrated angles extracted from the orders of diffraction.

4. Transfer matrix simulations

The theoretical simulations of the PS:DCJTB microcavity as a function of photon energy and viewing angle have been carried out by using the Transfer Matrix Method (TMM). [1] The simulated photonic structure consists of several pairs of alternated SiO_2 and TiO_2 layers, each one with a thickness of λ/4n, where λ=535nm is the central wavelength of the DBR and n is the refractive index of each layer. For the DBR layers, tabulated values of refractive indexes have been used [2], while we assumed an approximated refractive index of 1.7 for the polymeric spacer. It is worth noting that the low concentration of the dye (0.5%) allows us to neglect its contribution to the cavity spacer refractive index in our simulations. We considered semi-infinite layers for the top air space and the bottom glass substrate. The total cavity structure was: air (n=1)/(SiO_2/TiO_2) x8/cavity spacer/(TiO_2/SiO_2) x10.5/glass (n=1.5). We left the cavity spacer thickness as a free parameter of the model, finding the best match with the experimental data using a thickness of 202 nm.

5. Microscope configurations

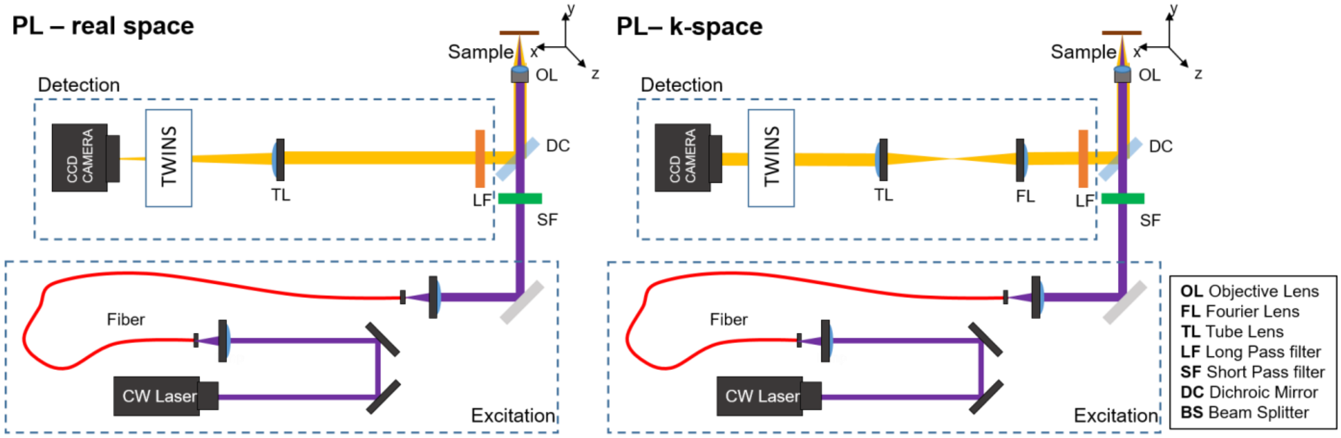

Figure S2. (Left) Schematics of the hyperspectral microscope setup in the configuration for PL measurements in real space. A fiber-coupled UV laser is used for the illumination, which is filtered out of the detection path by using a dichroic mirror and a long-pass filter. (Right) Schematics of the hyperspectral microscope setup in the configuration for PL measurements in k-space. In this case, a Fourier (Bertrand) lens (FL) is inserted in the detection path to image the back focal plane of the microscope objective.

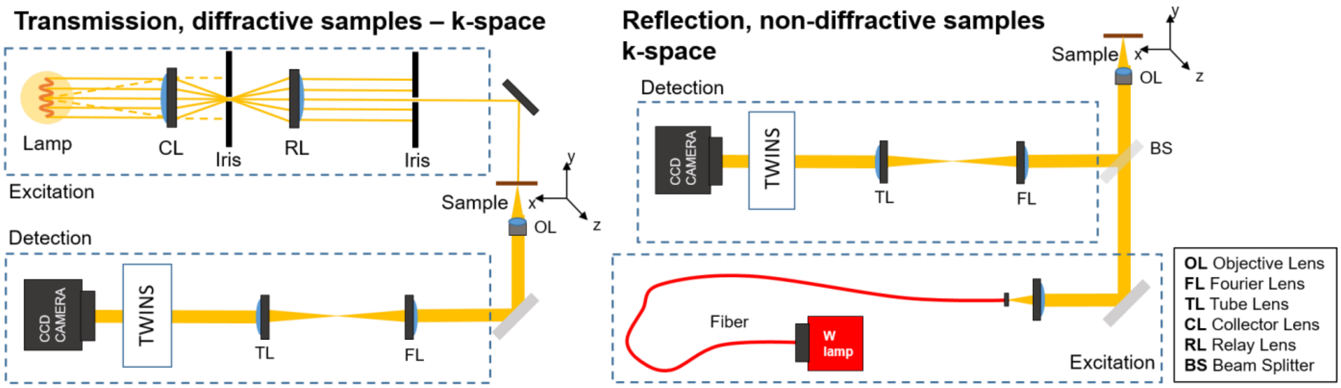

Figure S3. (Left) Schematics of the hyperspectral microscope setup configured for transmission measurements of diffractive samples in k-space. In order to get a nearly collimated illumination beam, we removed the condenser from the microscope built-in excitation path, closing both the field and the aperture irises. (Right) Schematics of the hyperspectral microscope setup in the configuration for reflection measurements in k-space for non-diffractive samples. A fiber-coupled Tungsten lamp is used for the illumination.

6. GaP nanodisks simulations

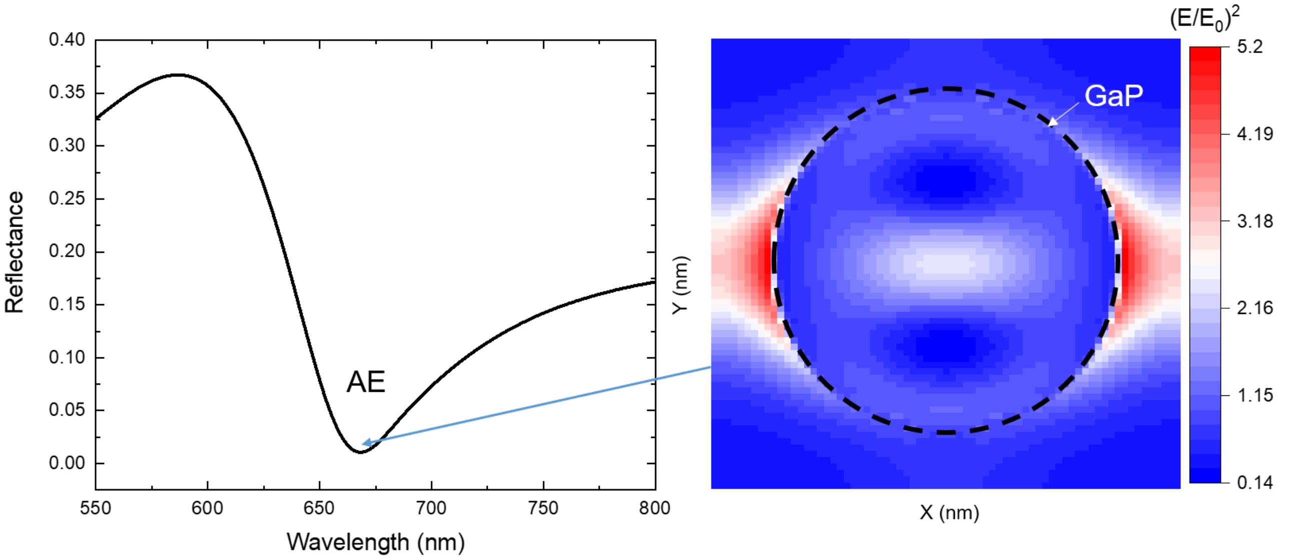

Figure S4. (Left) FDTD simulated reflectance of the GaP nanodisks metasurface excited at normal incidence, showing a clear dip at about 660nm, ascribed to the anapole excitation (AE). To avoid undesirable effects introduced by the dispersive substrate in the simulations under angular excitation, we simulated the nanodisks array without the presence of a substrate in free space. (Right) Top view of a GaP nanodisk with a color map of the simulated electric field intensity produced at the AE dip wavelength (monitored at the nanodisk half height), exhibiting the expected pattern of the field confined within the nanostructure ascribed to the anapole resonance.

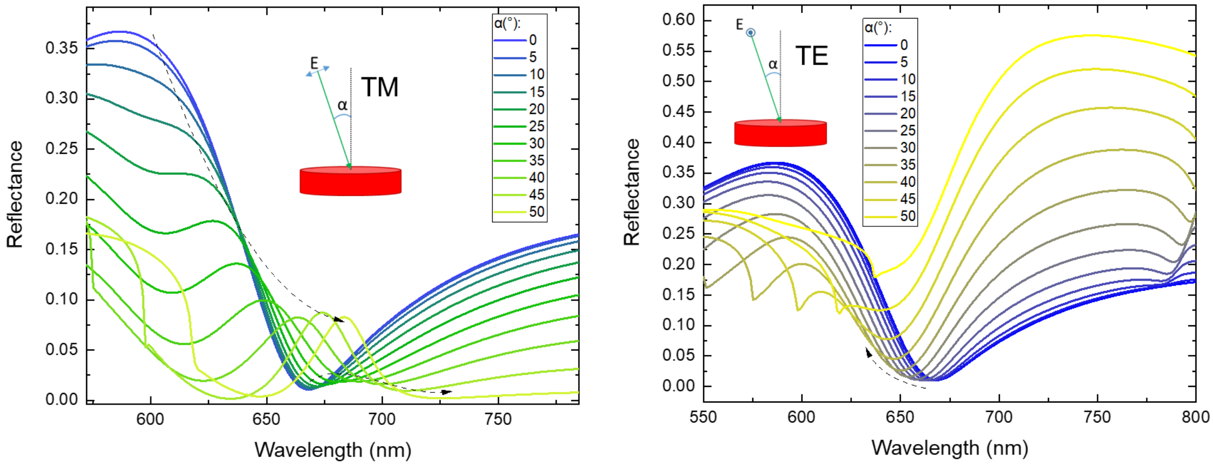

Figure S5. (Left) FDTD simulations of the reflectance of the GaP nanodisks metasurface as a function of the excitation angle for TM polarized light, showing a gradual redshift of the AE mode increasing the angle. (Right) FDTD simulation of the reflectance of the GaP nanodisks metasurface as a function of the angle for TE polarized light, showing a slight blueshift of the AE mode, gradually disappearing under increasing angles.

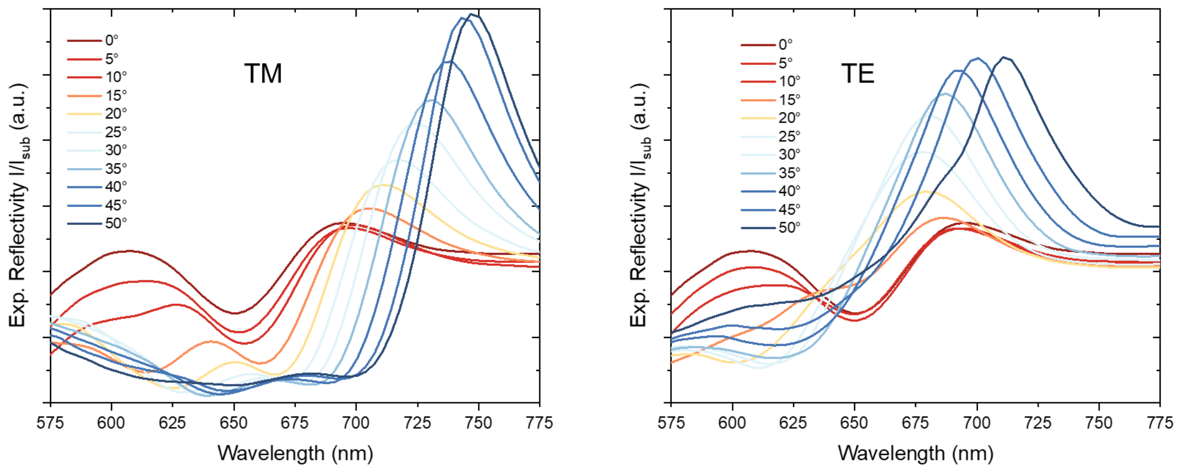

Figure S6. Experimental reflectivity (I/I$_{sub}$) spectra measured on the GaP nanodisks metasurface, extracted from the k-space hyperspectral image for different angles, for TM (Left) and TE (Right) polarized light. For both the polarizations, the AE mode angular dispersion shows a very good match with the simulated behaviour. The spectra are the same of Fig.5d, but plotted at precise angles in order to facilitate the comparison with the simulations.

7. GaP nanodisks raw k-space maps

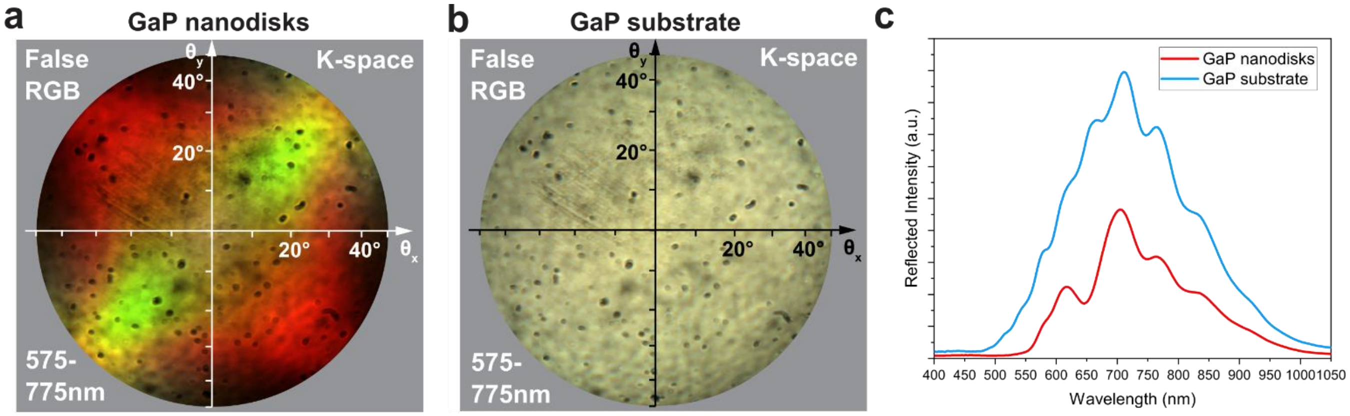

Figure S7. (a) False RGB image of the raw hyperspectral k-space reflected intensity measured on the GaP nanodisks, plotted in angular coordinates. (b) False RGB image of the raw hyperspectral k-space reflected intensity measured on the GaP substrate, plotted in angular coordinates. (c) Raw spectra extracted from the hyperspectral k-space images at zero angle for the nanodisks and the substrate, respectively.

References

1. P. Yeh, *Optical Waves in Layered Media* (Wiley-Interscience, 2005), Vol. 61.

2. E. D. Palik, *Handbook of Optical Constants of Solids* (Academic press, 1998), Vol. 3
